# Multidisciplinary attitudes towards LLM-structured oncologic CT reports: a prospective fixed-sequence questionnaire study

**DOI:** 10.1186/s40644-026-01137-2

**Published:** 2026-10-08

**Authors:** Felix Busch, Alexander W. Marka, Lilly F. Wingberg, Philipp Prucker, Su Hwan Kim, Quirin Strotzer, Christina Schmidt, Jacqueline Lammert, Kai J. Borm, Jan Peeken, Marcus R. Makowski, Lisa C. Adams, Keno K. Bressem

**Affiliations:** 1 Institute for Diagnostic and Interventional Radiology, TUM School of Medicine and Health, TUM University Hospital Rechts der Isar, Technical University of Munich, Munich, Germany; 2 Institute of Artificial Intelligence in Medicine and Healthcare, TUM School of Medicine and Health, TUM University Hospital Rechts der Isar, Munich, Germany; 3 Institute of Diagnostic and Interventional Neuroradiology, TUM School of Medicine and Health, TUM University Hospital Rechts der Isar, Technical University of Munich, Munich, Germany; 4 Department of Radiology, University Medical Center Regensburg, Regensburg, Germany; 5 Department of Radiology, Cambridge University Hospitals NHS Foundation Trust, Addenbrooke’s Hospital, Cambridge, England UK; 6 Department of Gynecology and Center for Hereditary Breast and Ovarian Cancer, TUM School of Medicine and Health, TUM University Hospital Rechts der Isar, Technical University of Munich, Munich, Germany; 7 Center for Personalized Medicine (ZPM), TUM School of Medicine and Health, TUM University Hospital Rechts der Isar, Technical University of Munich, Munich, Germany; 8 Department of Radiation Oncology, TUM School of Medicine and Health, TUM University Hospital Rechts der Isar, Technical University of Munich, Munich, Germany; 9 Bavarian Center for Cancer Research (BZKF), Technical University Munich, Munich, Germany; 10 Institute of Radiation Medicine, Helmholtz Zentrum München, Munich, Germany; 11 German Cancer Consortium, Partner Site Munich, Munich, Germany

**Keywords:** Tomography, X-ray computed, Neoplasms, Natural language processing, Medical records systems, computerized, Decision making

## Abstract

**Objectives:**

Large language models (LLMs) can automate the conversion of radiologic free text into structured reports, but multidisciplinary reader preferences for the resulting output remain insufficiently studied. This prospective, two-phase, fixed-sequence within-participant study evaluated reader assessments of LLM-structured versus conventional free-text oncologic CT reports.

**Material & Methods:**

A paired convenience sample of radiologists, radiology technologists, oncologists, and medical students was recruited between May and November 2025. In Phase 1, participants rated 5 free-text oncologic CT reports for readability and comprehension (3 items), oncologic information quality (5 items), and clinical utility (4 items) on a 9-point Likert scale. After a washout of at least 4 weeks, participants rated the LLM-structured version generated by Llama-4-Scout-17B-16E-Instruct.

**Results:**

Sixty-six paired participants (24 medical students, 22 radiologists, 12 radiology technologists, 8 oncologists) were included (mean [standard deviation] age, 30.5 [7.6] years; 37 [56.1%] female). Structured reports showed significantly higher odds of agreement for 11 of 12 items (odds ratio [OR] range, 1.70–4.81), with strongest effects for clinical utility (OR, 4.81; 95% CI, 3.24–7.15), information findability (OR, 4.31; 95% CI, 3.12–5.96), recommendation clarity (OR, 4.14; 95% CI, 2.95–5.83), and patient communication suitability (OR, 3.45; 95% CI, 2.59–4.60). Radiology technologists showed significantly higher ratings for all 12 items, radiologists for 6, students for 7, and oncologists for none.

**Conclusion:**

In this single-center paired sample, LLM-structured oncologic CT reports received higher reader ratings than conventional free-text reports across most evaluated dimensions, with the largest differences observed for information findability and clinical utility. These findings support further evaluation of radiologist-supervised LLM-assisted report structuring, including formal source-to-output validation and randomized multicenter assessment, before clinical implementation.

**Supplementary information:**

The online version contains supplementary material available at https://doi.org/10.1186/s40644-026-01137-2 .

## Introduction

Radiology reports are the primary communication tool between radiologists and referring clinicians and play a central role in diagnostic interpretation, treatment planning, and longitudinal disease assessment [1]. In oncologic imaging, this communicative function takes on added weight. Reports need to capture staging, treatment response, and actionable findings in a way that serves a multidisciplinary team, often with limited time and high clinical stakes [1–3].

Structured reporting has been proposed as a way to improve the consistency and quality of radiologic communication compared to conventional free-text formats [4, 5]. Studies have shown benefits in completeness, readability, and information retrieval, alongside higher satisfaction among referring physicians [1, 5–7]. In cancer imaging specifically, structured reports have been linked to better accessibility of critical findings, stronger support for treatment decisions, and clearer communication around staging and therapy response [2, 8–11].

Despite these possible advantages, structured reporting has not been widely implemented in standard radiology practice [12, 13]. Many radiologists see the value in principle, but in practice, concerns about rigid templates, oversimplification, and disruption to established workflows have slowed implementation [12, 14, 15]. There is also a methodological gap in the existing literature. Most studies have evaluated radiologist and referring clinician perspectives, while other groups involved in the day-to-day imaging workflow, technologists, trainees, broader multidisciplinary users, have received far less attention [1, 4, 5, 16–19].

Large language models (LLMs) may provide an effective method to enable structured reporting by automatically converting standard free-text reports into template-based formats while maintaining the original clinical content [20–22]. Preliminary studies have shown that LLMs excel in report structuring, summarization, and various text-oriented radiology workflows [23, 24]. Nonetheless, AI-assisted reporting raises significant concerns because of hallucinations, omissions, inconsistencies, and the necessity for human supervision prior to clinical deployment [20]. Moreover, although technical feasibility is increasingly being demonstrated, evidence on end-user acceptance of LLM-structured radiology reports remains limited, particularly in prospective multidisciplinary settings [25].

This study aims to evaluate the preferences of multidisciplinary readers, including radiologists, radiology technologists, oncologists, and medical students, towards the readability, comprehension, quality of oncological information and clinical usefulness of LLM-structured versus conventional free-text oncological CT reports, using a prospective, fixed-sequence questionnaire design.

## Methods

### Study design

This prospective, two-phase, fixed-sequence within-participant study compared multidisciplinary reader assessments of LLM-structured and conventional free-text oncologic CT reports. The study was designed to assess how readers evaluated structured reports after an initial assessment of the conventional reporting format. Ethical approval was obtained from the institutional review board of the Technical University of Munich (2024–590-S-CB) for non-patient-subject research, as the study involved synthetic radiology reports and a voluntary survey of clinical staff with no patient data. All participants provided informed consent for publication of their anonymized responses. This study is reported in accordance with the Strengthening the Reporting of Observational Studies in Epidemiology (STROBE) guideline.

### Setting and participants

A multidisciplinary, non-probability, convenience, paired sample was recruited between May and November 2025 at the Technical University of Munich. Invitations were distributed via institutional e-mail, with recipients encouraged to forward the invitation to eligible colleagues. Because the total number of individuals reached could not be determined, no response rate was calculated. In Phase 1, all participants evaluated five conventional free-text reports, one for each cancer domain. After a minimum washout interval of four weeks, the same participants evaluated the corresponding LLM-structured versions using the same questionnaire. Sequence allocation and counterbalancing were not performed. Eligible participants were radiologists, radiology technologists, oncologists, and medical students completing both phases within the specified timeframe and after the minimum washout interval. Duplicate or incomplete submissions were excluded from evaluation. The number of participants lost to follow-up between Phases 1 and 2 was recorded.

### Report development

Five cancer-specific structured English reporting templates for CT examinations of colorectal, breast, prostate, hepatocellular carcinoma, and lung malignancies were developed by two bilingual radiologists with 2 and 5 years of experience in cross-sectional imaging and finalized in consensus with a board-certified radiologist with 10 years of experience. All templates followed a common oncologic reporting framework comprising clinical history and indication, imaging technique, comparison with previous imaging, TNM-relevant findings, additional findings, and impression (see Supplemental Material, eTable 1).

Disease-specific items were mapped to the staging and reporting standards applicable when the templates were finalized and the study was initiated, including the eighth edition of the AJCC TNM classification [26] and LI-RADS v2018 for hepatocellular carcinoma [27]. Additional entity-specific fields included PSA values and ISUP grade groups for prostate cancer. The templates were finalized before participant recruitment and remained unchanged throughout data collection.

Where applicable, the templates recorded prior or current treatment, the clinical indication, comparison with previous imaging, lesion-specific interval change, and overall treatment response. Information unavailable in the source report was labelled ‘N/A’ rather than inferred. The templates served as organizational frameworks for information contained in the source CT reports and were not intended to determine whether CT was the optimal or sufficient imaging modality for a particular clinical question.

The prostate-cancer template explicitly states that CT has limited sensitivity for local T staging and identifies multiparametric MRI as the preferred modality for local staging and PSMA PET/CT as the preferred modality for detecting occult nodal or distant metastatic disease. The CT-specific sections focus on findings assessable on oncologic CT, including gross local extension, lymphadenopathy, osseous and visceral metastatic disease, and interval change.

For each cancer type, corresponding unstructured English free-text reports were drafted by the same radiologists in consensus, using phrasing and terminology reflective of routine clinical practice.

Each free-text report was converted using Llama-4-Scout-17B-16E-Instruct (Meta Platforms, Inc., Menlo Park, CA, USA), deployed locally in an institutional Ollama environment. The model received the source report as text only. No image data were provided. Temperature was set to 0 to minimize sampling-related output variability. No additional inference-parameter tuning was performed, and all remaining inference parameters were retained at the default settings of the local Ollama runtime. The complete conversion prompt and all disease-specific templates are provided in eTable 1.

The conversion prompt instructed the model to reorganize each source report according to the corresponding disease-specific template while preserving the source content. Information that was not contained in the source report was to be marked as ‘N/A’ rather than inferred. Each template also included an internal consistency checklist.

Before survey administration, each structured report was compared separately with its source report by each of the two radiologists involved in preparing the study materials. Their assessments were subsequently reconciled in consensus, with any remaining disagreement referred to the senior board-certified radiologist. Only consensus-approved reports were included in the survey. This author-led review constituted quality control of the survey materials and was not designed as a formal quantitative accuracy study.

### Survey design and variables

The questionnaire comprised 12 items rated on a 9-point Likert scale (1 = very poor to 9 = excellent), organized into 3 dimensions. The readability and comprehension dimension included readability (Q1), clarity of formulations (Q2), and ease of finding relevant information (Q3). The oncologic information quality dimension included clarity of oncologic information (Q4), structure and organization (Q5), comprehensibility (Q6), completeness (Q7), and precision (Q8). The clinical utility dimension included clinical usefulness (Q9), clinical decision support (Q10), clarity of actionable recommendations (Q11), and suitability for patient communication (Q12). The demographics section captured self-reported gender, age, professional affiliation, and professional experience in years for radiologists, radiology technologists, and oncologists. Medical students reported their current year of study rather than professional experience. The survey was administered in English using Google Forms. The participant-facing survey title and instructions described the project neutrally as an evaluation of oncologic radiology reports. Participants were not informed that the study compared reporting formats, that a second phase using a different format would follow, or that the structured reports had been generated using an LLM. The reports were evaluated in separate phases and were not presented side by side. Because the structural differences were visible, participants could not be blinded to the report format. Within each phase, the five reports were presented in the same fixed order to all participants, and this order was retained across the two phases. Report order was not randomized. Participants completed the questionnaires remotely and asynchronously and were instructed to evaluate the reports independently. No group-evaluation sessions or discussions were conducted by the study team.

### Statistical analysis

Statistical analyses were performed in Python 3.12 using SciPy 1.14, statsmodels 0.14, and pandas 2.2. Descriptive survey results were summarized as means with standard deviations for the total cohort and stratified by expertise group. Paired Wilcoxon signed-rank tests were used to compare person-level mean ratings between free-text and structured formats within each subgroup. A generalized linear mixed model (GLMM) was fitted independently for each of the 12 survey items. Ratings were dichotomized into agreement (score ≥ 6) versus nonagreement (score < 6). A random intercept for participant was included to account for the paired, fixed-sequence within-participant design and individual rating tendencies. Fixed effects included report format (structured vs free-text), expertise group (reference: students), age (z-standardized), an experience or training-stage covariate (z-standardized), and cancer domain (reference: colorectal cancer). Professional experience was used for radiologists, radiology technologists, and oncologists, whereas current year of study was recorded for medical students. Mixed model results were reported as odds ratios (OR) with 95% confidence intervals (CI). A two-sided *p* < 0.05 was considered statistically significant. Analyses followed a complete-case paired approach. Only participants with valid responses in both phases were included in the descriptive format comparisons, paired tests, and generalized linear mixed models. Phase 1 ratings from participants who did not complete Phase 2 were excluded from the report-format comparisons, and missing Phase 2 outcomes were not imputed.

## Results

### Participants

**Fig. 1:**
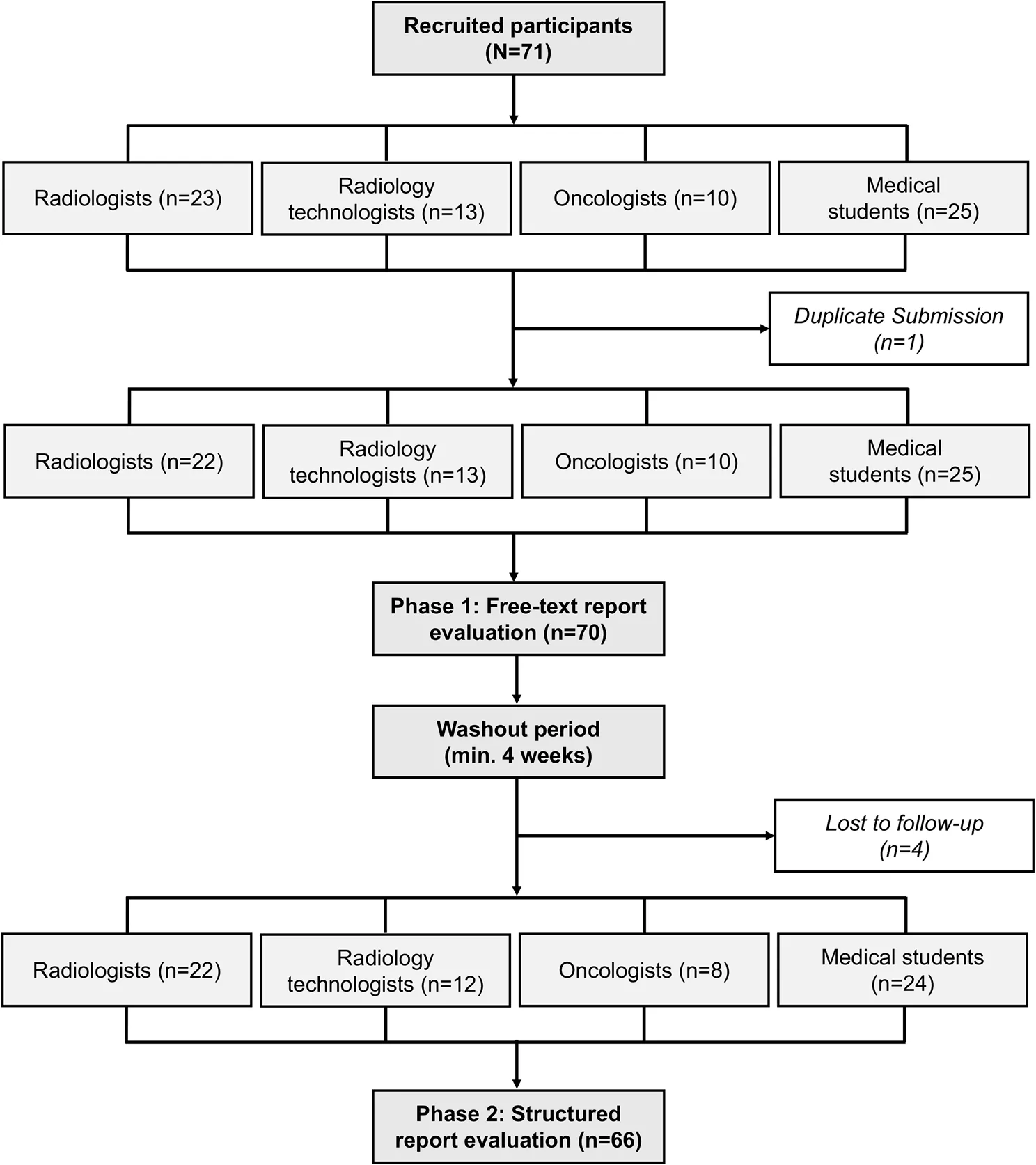
Participant flow diagram illustrating the total number of participants and the number per expertise group at each stage

Phase 1 yielded 71 submissions. After exclusion of one duplicate submission, 70 unique participants remained eligible for follow-up. Sixty-six participants completed Phase 2 and formed the paired analysis cohort, while four participants were lost to follow-up. The overall attrition rate after exclusion of the duplicate was 5.7% (see participant flow diagram in Fig. 1). Characteristics of the participants lost to follow-up are reported in eTable 2. The 66 paired participants comprised 24 medical students (36.4%), 22 radiologists (33.3%), 12 radiology technologists (18.2%), and 8 oncologists (12.1%). The participants lost to follow-up comprised one radiology technologist, two oncologists, and one medical student. Their Phase 1 ratings were not included in the report-format comparisons. The median washout period between both phases was 56 days (interquartile range, 38–76 days). Radiologists reported a mean of 2.8 years of professional experience (SD, 2.0), and oncologists reported a mean of 5.1 years (SD, 2.6). Radiology technologists reported a mean of 19.6 years of professional experience (SD, 11.6). For medical students, the corresponding value represented the current year of study, with a mean of 5.0 years (SD, 1.7). Sociodemographic characteristics for the overall study population and by expertise group are shown in Table 1.

**Table 1:** Basic characteristics of the overall cohort and of each expertise group

Characteristic	Overall	Radiologists	Technologists	Oncologists	Students
No. of participants (%)	66 (100)	22 (33.3)	12 (18.2)	8 (12.1)	24 (36.4)
Gender, no. (%)					
Female	37 (56.1)	6 (27.3)	10 (83.3)	4 (50.0)	17 (70.8)
Male	29 (43.9)	16 (72.7)	2 (16.7)	4 (50.0)	7 (29.2)
Age, mean (SD), y	30.5 (7.6)	29.6 (2.7)	40.8 (10.6)	34.2 (4.3)	24.8 (1.5)
Experience^†^, mean (SD), y	6.9 (7.9)	2.8 (2.0)	19.6 (11.6)	5.1 (2.6)	5.0 (1.7)

**Note:** Abbreviation: SD, standard deviation. ^†^ For medical students, values reflect current year of study rather than years of professional experience

### Readability and comprehension

**Fig. 2:**
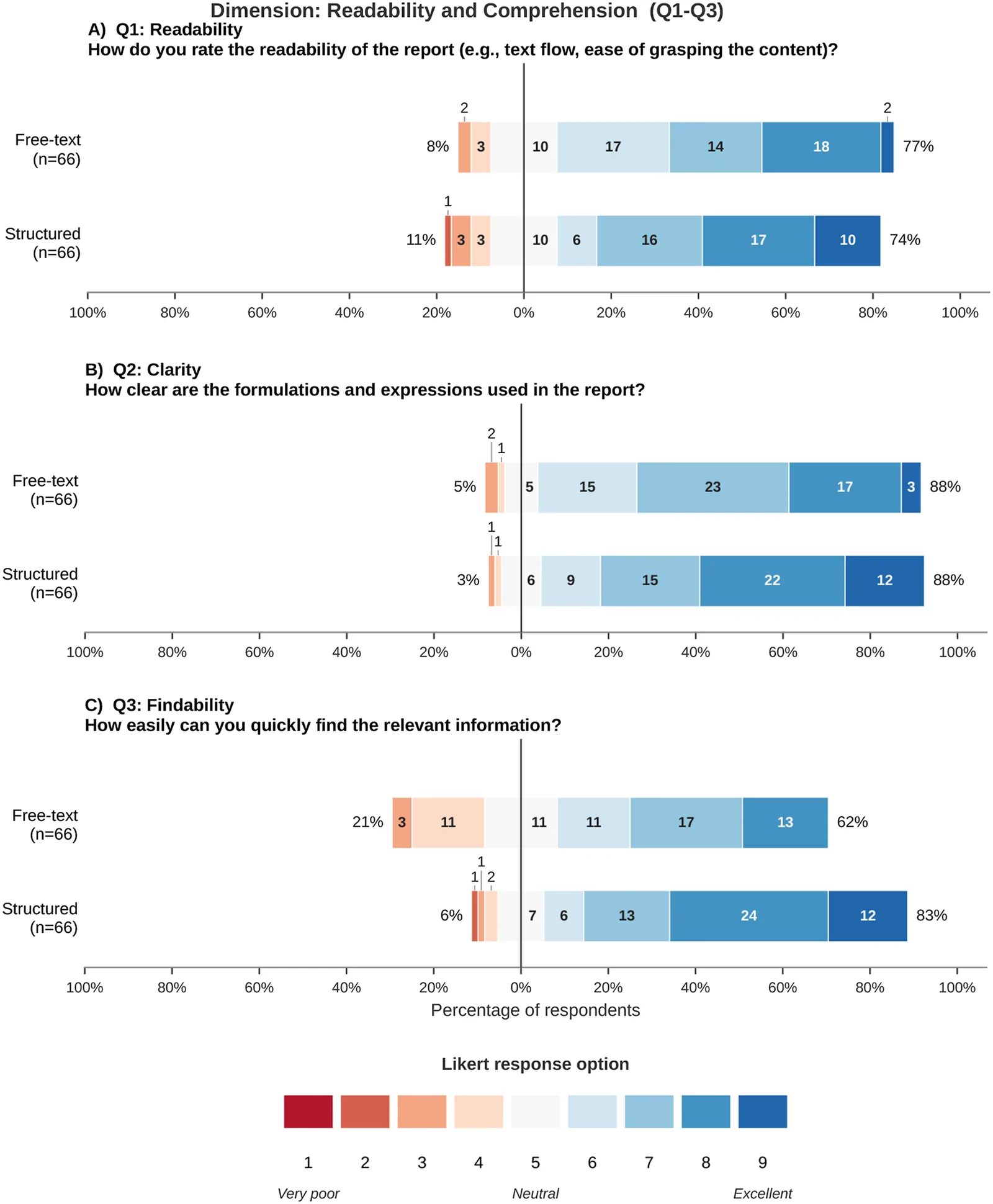
Gantt diagrams illustrating item responses for the readability and comprehension domain for the overall cohort (*n* = 66): **A**) readability (Q1), **B**) clarity (Q2), **C**) findability (Q3)

Descriptive survey results for all 12 items stratified by expertise group are presented in Table 2. GLMM estimates for each item and variable are provided in Table 3. Readability (Q1) was not significantly altered between reporting methods (OR, 1.15; 95% CI, 0.86–1.54; *p* = 0.35). Clarity of formulations (Q2) improved from 6.8 (1.6) to 7.3 (1.6) (OR, 1.70; 95% CI, 1.19–2.43; *p* = 0.004). The strongest effect was observed for findability (Q3), where mean ratings increased from 6.0 (2.0) to 7.1 (1.8) (OR, 4.31; 95% CI, 3.12–5.96; *p* < 0.001). Technologists were the only group who showed significant improvements for all 3 items, while radiologists and students showed significant improvement for findability (Q3). Figure 2 provides Gantt diagrams for the readability and comprehension domain, illustrating the responses of the overall cohort. Figures S1–S4 provide the corresponding Gantt diagrams for each expertise group.

**Table 2:** Survey results by item, format, and expertise group

Variable	Overall	Radiologists	Technologists	Oncologists	Students
**Q1: Readability**					
Free-text, mean (SD)	6.5 (1.8)	6.5 (1.7)	6.6 (1.9)	6.8 (1.7)	6.5 (1.7)
Structured, mean (SD)	6.8 (1.9)	6.4 (1.7)	7.7 (1.9)	6.5 (2.1)	6.8 (1.9)
*p*-value	0.172	0.884	**0.030**	0.844	0.345
**Q2: Clarity**					
Free-text, mean (SD)	6.8 (1.6)	6.8 (1.6)	6.8 (1.7)	6.7 (1.7)	6.8 (1.5)
Structured, mean (SD)	7.3 (1.6)	7.2 (1.5)	7.8 (1.8)	7.3 (1.8)	7.1 (1.6)
*p*-value	**0.017**	0.454	**0.025**	0.641	0.247
**Q3: Findability**					
Free-text, mean (SD)	6.0 (2.0)	5.8 (2.0)	6.0 (2.1)	6.9 (1.7)	5.8 (2.0)
Structured, mean (SD)	7.1 (1.8)	7.2 (1.4)	7.7 (2.0)	7.0 (2.1)	6.9 (2.0)
*p*-value	**<0.001**	**0.004**	**0.009**	0.867	**0.013**
**Q4: Oncologic clarity**					
Free-text, mean (SD)	6.5 (1.9)	6.5 (2.0)	6.4 (2.1)	6.9 (1.8)	6.4 (1.8)
Structured, mean (SD)	7.1 (1.8)	7.2 (1.7)	7.5 (1.9)	7.0 (1.9)	6.9 (1.8)
*p*-value	**0.003**	**0.049**	**0.040**	0.969	0.126
**Q5: Structure/organization**					
Free-text, mean (SD)	6.3 (2.0)	6.0 (2.1)	6.2 (2.0)	6.9 (1.8)	6.4 (1.8)
Structured, mean (SD)	7.3 (1.7)	7.2 (1.7)	7.6 (1.7)	7.1 (1.9)	7.3 (1.6)
*p*-value	**<0.001**	**0.013**	**0.005**	0.844	**0.031**
**Q6: Comprehensibility**					
Free-text, mean (SD)	6.5 (1.8)	6.6 (1.9)	6.5 (1.9)	6.9 (1.9)	6.4 (1.7)
Structured, mean (SD)	7.2 (1.6)	7.2 (1.5)	7.7 (1.7)	7.3 (1.6)	7.0 (1.7)
*p*-value	**<0.001**	0.127	**0.005**	0.820	**0.024**
**Q7: Completeness**					
Free-text, mean (SD)	6.5 (2.0)	6.5 (2.2)	6.6 (1.9)	6.9 (2.0)	6.4 (1.8)
Structured, mean (SD)	7.2 (1.7)	7.2 (1.6)	7.5 (1.7)	7.8 (1.3)	6.8 (1.7)
*p*-value	**<0.001**	0.062	**0.045**	0.398	0.094
**Q8: Precision**					
Free-text, mean (SD)	6.3 (2.0)	6.2 (2.1)	6.2 (2.0)	6.6 (2.1)	6.3 (1.7)
Structured, mean (SD)	7.0 (1.7)	7.0 (1.8)	7.4 (1.7)	7.3 (1.7)	6.7 (1.7)
*p*-value	**<0.001**	**0.025**	**0.007**	0.438	0.144
**Q9: Clinical utility**					
Free-text, mean (SD)	6.6 (2.0)	6.4 (2.2)	6.4 (2.3)	7.2 (2.0)	6.7 (1.5)
Structured, mean (SD)	7.4 (1.5)	7.5 (1.3)	7.6 (1.8)	8.0 (0.9)	7.1 (1.7)
*p*-value	**<0.001**	**0.012**	**0.033**	0.383	**0.026**
**Q10: Clinical decision support**					
Free-text, mean (SD)	6.9 (1.8)	7.1 (1.6)	6.3 (2.4)	7.5 (1.8)	6.8 (1.5)
Structured, mean (SD)	7.5 (1.5)	7.7 (1.2)	7.6 (1.6)	8.1 (0.9)	7.1 (1.6)
*p*-value	**<0.001**	0.052	**0.011**	0.781	**0.044**
**Q11: Recommendation clarity**					
Free-text, mean (SD)	5.9 (2.2)	5.9 (2.1)	5.5 (2.3)	7.5 (1.8)	5.7 (2.1)
Structured, mean (SD)	6.9 (1.8)	7.0 (1.6)	7.4 (1.8)	7.9 (0.9)	6.2 (2.0)
*p*-value	**<0.001**	**0.019**	**0.001**	0.945	**0.006**
**Q12: Patient communication**					
Free-text, mean (SD)	4.9 (2.2)	4.5 (2.0)	5.2 (2.3)	5.5 (2.5)	4.8 (2.2)
Structured, mean (SD)	5.9 (2.3)	5.2 (2.3)	7.0 (2.0)	5.9 (2.6)	6.0 (2.1)
*p*-value	**<0.001**	0.148	**<0.001**	0.641	**0.012**

**Note:** Bold *p*-values indicate *p* < 0.05. Abbreviations: SD, standard deviation

**Table 3:** Logistic generalized linear mixed model results by variable and survey item

Variable	OR (95% CI)	*p***-value**
**Q1: Readability**		
Structured reports^a^	1.15 (0.86–1.54)	0.347
Radiologist^b^	1.14 (0.81–1.58)	0.453
Technologist^b^	1.42 (0.80–2.52)	0.229
Oncologist^b^	1.18 (0.68–2.03)	0.563
Age	0.83 (0.64–1.07)	0.155
Years of experience	1.71 (1.30–2.26)	**<0.001**
Breast^c^	1.32 (0.83–2.10)	0.233
Prostate^c^	2.17 (1.30–3.60)	**0.003**
Hepatocellular carcinoma^c^	0.75 (0.49–1.15)	0.190
Lung^c^	1.01 (0.65–1.58)	0.957
**Q2: Clarity**		
Structured reports^a^	1.70 (1.19–2.43)	**0.004**
Radiologist^b^	2.04 (1.35–3.08)	**<0.001**
Technologist^b^	3.14 (1.58–6.21)	**0.001**
Oncologist^b^	1.68 (0.90–3.14)	0.106
Age	0.62 (0.47–0.82)	**<0.001**
Years of experience	1.48 (1.11–1.98)	**0.008**
Breast^c^	0.79 (0.46–1.36)	0.393
Prostate^c^	1.11 (0.61–1.99)	0.739
Hepatocellular carcinoma^c^	0.35 (0.22–0.57)	**<0.001**
Lung^c^	0.59 (0.35–0.98)	**0.042**
**Q3: Findability**		
Structured reports^a^	4.31 (3.12–5.96)	**<0.001**
Radiologist^b^	1.52 (1.08–2.13)	**0.017**
Technologist^b^	2.59 (1.50–4.47)	**<0.001**
Oncologist^b^	2.10 (1.15–3.82)	**0.015**
Age	0.86 (0.68–1.10)	0.227
Years of experience	1.20 (0.94–1.54)	0.139
Breast^c^	2.10 (1.32–3.35)	**0.002**
Prostate^c^	3.96 (2.36–6.66)	**<0.001**
Hepatocellular carcinoma^c^	1.60 (1.02–2.50)	**0.040**
Lung^c^	1.18 (0.76–1.82)	0.463
**Q4: Oncologic clarity**		
Structured reports^a^	1.89 (1.36–2.62)	**<0.001**
Radiologist^b^	1.88 (1.29–2.74)	**<0.001**
Technologist^b^	1.50 (0.85–2.64)	0.160
Oncologist^b^	1.77 (0.96–3.23)	0.065
Age	0.81 (0.62–1.04)	0.104
Years of experience	1.51 (1.15–1.98)	**0.003**
Breast^c^	0.68 (0.42–1.09)	0.107
Prostate^c^	0.87 (0.53–1.42)	0.570
Hepatocellular carcinoma^c^	0.76 (0.47–1.24)	0.275
Lung^c^	0.72 (0.45–1.16)	0.176
**Q5: Structure/organization**		
Structured reports^a^	3.10 (2.20–4.37)	**<0.001**
Radiologist^b^	1.36 (0.95–1.95)	0.092
Technologist^b^	0.74 (0.42–1.28)	0.276
Oncologist^b^	1.83 (0.97–3.45)	0.060
Age	0.77 (0.58–1.01)	0.063
Years of experience	2.09 (1.55–2.81)	**<0.001**
Breast^c^	1.24 (0.75–2.04)	0.398
Prostate^c^	1.09 (0.67–1.77)	0.726
Hepatocellular carcinoma^c^	0.97 (0.60–1.55)	0.884
Lung^c^	0.91 (0.57–1.46)	0.694
**Q6: Comprehensibility**		
Structured reports^a^	2.14 (1.51–3.04)	**<0.001**
Radiologist^b^	2.00 (1.35–2.97)	**<0.001**
Technologist^b^	1.22 (0.66–2.25)	0.534
Oncologist^b^	1.94 (1.03–3.66)	**0.040**
Age	0.72 (0.54–0.97)	**0.031**
Years of experience	2.16 (1.57–2.98)	**<0.001**
Breast^c^	1.27 (0.75–2.15)	0.376
Prostate^c^	1.48 (0.86–2.54)	0.160
Hepatocellular carcinoma^c^	0.96 (0.58–1.59)	0.875
Lung^c^	0.84 (0.52–1.38)	0.497
**Q7: Completeness**		
Structured reports^a^	2.76 (1.93–3.93)	**<0.001**
Radiologist^b^	1.58 (1.08–2.30)	**0.018**
Technologist^b^	1.10 (0.62–1.94)	0.742
Oncologist^b^	3.96 (1.81–8.65)	**<0.001**
Age	0.84 (0.63–1.12)	0.235
Years of experience	1.91 (1.42–2.57)	**<0.001**
Breast^c^	0.70 (0.43–1.15)	0.159
Prostate^c^	0.80 (0.48–1.32)	0.384
Hepatocellular carcinoma^c^	1.06 (0.62–1.80)	0.833
Lung^c^	0.66 (0.40–1.07)	0.094
**Q8: Precision**		
Structured reports^a^	2.60 (1.88–3.60)	**<0.001**
Radiologist^b^	1.87 (1.30–2.70)	**<0.001**
Technologist^b^	1.65 (0.96–2.83)	0.073
Oncologist^b^	2.24 (1.21–4.15)	**0.011**
Age	0.82 (0.64–1.05)	0.124
Years of experience	1.54 (1.19–2.00)	**0.001**
Breast^c^	0.59 (0.38–0.92)	**0.020**
Prostate^c^	0.92 (0.57–1.47)	0.718
Hepatocellular carcinoma^c^	1.17 (0.72–1.91)	0.532
Lung^c^	0.92 (0.57–1.47)	0.718
**Q9: Clinical utility**		
Structured reports^a^	4.81 (3.24–7.15)	**<0.001**
Radiologist^b^	0.95 (0.64–1.41)	0.808
Technologist^b^	1.07 (0.58–1.98)	0.833
Oncologist^b^	2.82 (1.21–6.61)	**0.017**
Age	1.11 (0.81–1.51)	0.512
Years of experience	1.22 (0.89–1.66)	0.214
Breast^c^	1.43 (0.81–2.51)	0.219
Prostate^c^	0.96 (0.56–1.63)	0.880
Hepatocellular carcinoma^c^	1.12 (0.65–1.93)	0.688
Lung^c^	0.83 (0.49–1.40)	0.482
**Q10: Clinical decision support**		
Structured reports^a^	2.64 (1.75–3.98)	**<0.001**
Radiologist^b^	3.46 (2.12–5.67)	**<0.001**
Technologist^b^	0.66 (0.36–1.19)	0.163
Oncologist^b^	4.53 (1.85–11.08)	**<0.001**
Age	0.72 (0.52–0.98)	**0.039**
Years of experience	2.09 (1.50–2.90)	**<0.001**
Breast^c^	0.75 (0.42–1.35)	0.339
Prostate^c^	0.90 (0.50–1.64)	0.743
Hepatocellular carcinoma^c^	1.00 (0.54–1.83)	0.989
Lung^c^	0.54 (0.31–0.93)	**0.028**
**Q11: Recommendation clarity**		
Structured reports^a^	4.14 (2.95–5.83)	**<0.001**
Radiologist^b^	1.71 (1.17–2.50)	**0.006**
Technologist^b^	1.46 (0.87–2.44)	0.153
Oncologist^b^	9.58 (3.79–24.22)	**<0.001**
Age	1.24 (0.99–1.56)	0.067
Years of experience	0.78 (0.62–0.98)	**0.033**
Breast^c^	0.28 (0.17–0.46)	**<0.001**
Prostate^c^	0.50 (0.30–0.83)	**0.007**
Hepatocellular carcinoma^c^	0.50 (0.30–0.83)	**0.007**
Lung^c^	0.70 (0.42–1.18)	0.182
**Q12: Patient communication**		
Structured reports^a^	3.45 (2.59–4.60)	**<0.001**
Radiologist^b^	0.82 (0.59–1.14)	0.238
Technologist^b^	5.57 (3.26–9.52)	**<0.001**
Oncologist^b^	2.86 (1.50–5.45)	**0.001**
Age	0.51 (0.40–0.64)	**<0.001**
Years of experience	1.12 (0.89–1.42)	0.322
Breast^c^	1.80 (1.15–2.82)	**0.011**
Prostate^c^	2.35 (1.49–3.70)	**<0.001**
Hepatocellular carcinoma^c^	1.45 (0.93–2.28)	0.104
Lung^c^	2.11 (1.34–3.32)	**0.001**

**Note:** Bold *p*-values indicate *p* < 0.05. Abbreviations: OR, odds ratio; CI, confidence interval. Reference categories: ^a^ free-text format, ^b^ student group, ^c^ colorectal cancer domain

### Oncologic information quality

**Fig. 3:**
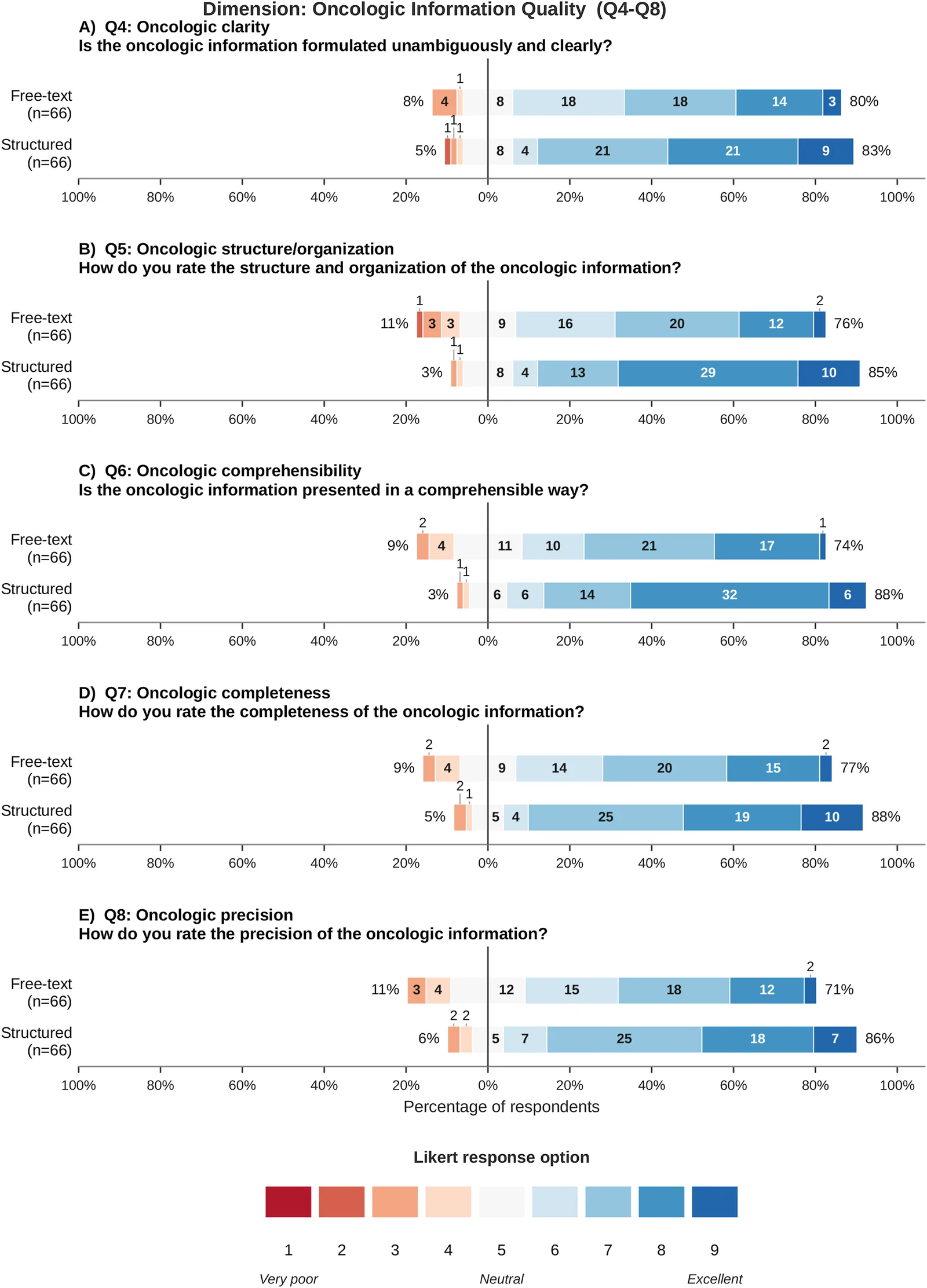
Gantt diagrams illustrating item responses for the oncologic information quality domain for the overall cohort (*n* = 66): **A**) oncologic clarity (Q4), **B**) oncologic structure/organization (Q5), **C**) oncologic comprehensibility (Q6), **D**) oncologic completeness (Q7), **E**) oncologic precision (Q8)

Structure and organization (Q5) showed the largest effect in this dimension, with mean ratings increasing from 6.3 (2.0) to 7.3 (1.7) (OR, 3.10; 95% CI, 2.20–4.37), followed by completeness (Q7; OR, 2.76; 95% CI, 1.93–3.93), precision (Q8; OR, 2.60; 95% CI, 1.88–3.60), comprehensibility (Q6; OR, 2.14; 95% CI, 1.51–3.04), and oncologic clarity (Q4; OR, 1.89; 95% CI, 1.36–2.62; all *p* < 0.001). Among expertise groups, technologists showed significant paired improvements for all 5 items. Radiologists showed significant improvements for 3 items (Q4, Q5, Q8), while students reached significance for 2 items (Q5, Q6). Figure 3 provides Gantt diagrams for the oncologic information quality domain, illustrating the responses of the overall cohort. Figures S5–S8 provide the corresponding Gantt diagrams for each expertise group.

### Clinical utility

**Fig. 4:**
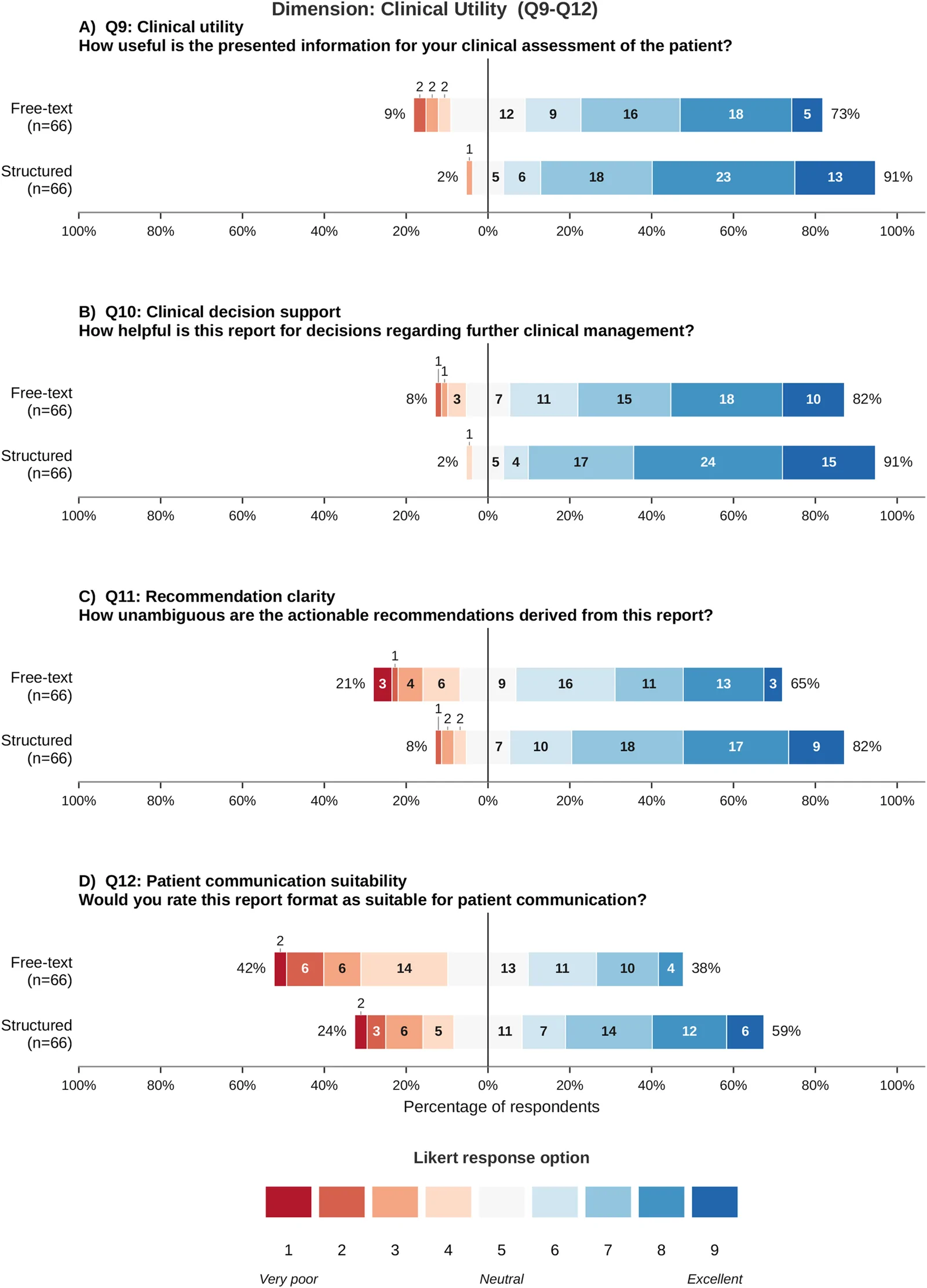
Gantt diagrams illustrating item responses for the clinical utility domain for the overall cohort (*n* = 66): **A**) clinical utility (Q9), **B**) clinical decision support (Q10), **C**) recommendation clarity (Q11), **D**) patient communication suitability (Q12)

Clinical usefulness (Q9) showed the highest odds ratio of all 12 items, with mean ratings increasing from 6.6 (2.0) to 7.4 (1.5) (OR, 4.81; 95% CI, 3.24–7.15), followed by recommendation clarity (Q11) (OR, 4.14; 95% CI, 2.95–5.83), suitability for patient communication (Q12) (OR, 3.45; 95% CI, 2.59–4.60), and clinical decision support (Q10) (OR, 2.64; 95% CI, 1.75–3.98; all *p* < 0.001). Technologists and students showed significant improvements for all 4 items. Radiologists showed significant improvements for Q9 and Q11. Figure 4 provides Gantt diagrams for the clinical utility domain, illustrating the responses of the overall cohort. Figures S9–S12 provide the corresponding Gantt diagrams for each expertise group.

### Covariates

Expertise group showed variable effects (see Table 3). For example, oncologists had significantly higher baseline odds of agreement than students for completeness (Q7; OR, 3.96; 95% CI, 1.81–8.65), recommendation clarity (Q11; OR, 9.58; 95% CI, 3.79–24.22), and clinical decision support (Q10; OR, 4.53; 95% CI, 1.85–11.08). Technologists rated patient communication (Q12) higher than students (OR, 5.57; 95% CI, 3.26–9.52; all *p* < 0.001). Cancer domain effects were variable across items. For example, prostate reports received higher findability ratings than colorectal (Q3; OR, 3.96; 95% CI, 2.36–6.66), while breast reports received lower recommendation clarity ratings (Q11; OR, 0.28; 95% CI, 0.17–0.46; both *p* < 0.001). Experience or training stage was positively associated with agreement for 8 of the 12 items, with odds ratios ranging from 1.48 to 2.16 (all *p* ≤ 0.008), while age showed an inverse association with agreement in 4 items (ORs, 0.51–0.72; all *p* ≤ 0.039). These results indicate that reader background influenced the ratings, while the report-format associations were estimated after adjustment for expertise group, age, experience or training stage, and cancer domain.

## Discussion

In this prospective fixed-sequence within-participant study of 66 multidisciplinary readers, LLM-structured oncologic CT reports received higher ratings than conventional free-text reports across nearly all evaluated dimensions, with significantly higher odds of agreement for 11 of 12 questionnaire items. The most pronounced effects were seen for clinical usefulness, ease of finding information, clarity of recommendations, and suitability for patient communication. Readability, by contrast, showed little difference between formats.

Prior work has consistently shown that structured reporting improves report quality relative to conventional narrative formats. Schwartz et al. found significantly higher ratings for both content satisfaction and clarity in structured body CT reports at a tertiary cancer center, with content scores rising from 7.61 to 8.33 and clarity scores from 7.45 to 8.25 [1], and comparable improvements in completeness and information extraction have been described across a range of structured reporting settings [4–7]. What distinguishes our findings, however, is where the gains were largest. Rather than global readability, the strongest improvements concerned functions that matter most in real-world oncologic care, such as the retrieval of key findings, clearer management recommendations, and perceived clinical utility. The implication is that structured formatting may matter less for how smoothly a report reads and more for how reliably a busy clinician can extract what they need from it. Earlier studies have made a related point, noting that structured reports can enhance accessibility of key information and physician satisfaction even when not every quality dimension improves [1, 28].

Our results fit well with prior evidence that structured reporting is especially valuable in cancer imaging, where reports must convey disease burden, interval change, and management-relevant findings in a clinically actionable way. For example, Weber et al. showed that referring oncologists rated structured oncology follow-up reports significantly better than conventional ones across all quality domains, with mean overall scores of 1.98 ± 1.22 vs. 3.05 ± 1.93 and a higher proportion of positive ratings (89% vs. 67%) [11]. Nörenberg et al. found that structured rectal cancer MRI reports enabled definite clinical decision-making in 96% of cases compared with 60% for free-text reports, and were considered sufficient for surgical planning in 94% vs. 38% [2]. Brook et al. similarly demonstrated that structured pancreatic CT reports contained substantially more key staging features (10.6 ± 0.9 vs. 7.3 ± 2.1) and more often provided adequate information for surgical planning [8]. Across these studies and our own, the added value of structured reporting is most evident when imaging findings directly inform staging, treatment planning, and multidisciplinary decision-making, as is the case in oncologic CT reporting.

One feature of our study worth highlighting is the inclusion of radiology technologists as a distinct reader group. Most structured reporting studies have compared radiologists and referring clinicians [1, 16, 17, 19], and technologist perspectives have received little systematic attention in this literature. The strong improvements we observed in this subgroup may reflect the value of clear, predictable report organization for readers who work closely with imaging workflows but are less practiced at navigating the variability of free-text narrative reports. The more moderate gains among radiologists, by contrast, may reflect their greater familiarity with narrative reports and capacity to compensate for structural inconsistency, which is also supported by prior survey data. For example, Kelsch et al. found that while expanded structured reporting was the most preferred style overall, radiologists more often preferred unstructured reporting than non-radiologists did (41% vs. 51%) [16]. This discrepancy suggests that the benefits of structured reporting may be greatest for downstream report users rather than for the radiologists who produce them, and underscores the importance of evaluating preferences across the full range of clinical stakeholders.

By contrast, oncologists did not show any significant paired improvements, most likely due to the limited sample size. In addition, oncologists gave relatively favorable ratings to the free-text reports at baseline, particularly for completeness, recommendation clarity, and decision support, which inevitably constrained how much improvement could be detected. Whether this pattern reflects genuine satisfaction with conventional reporting or a familiarity effect cannot be determined from the present data. However, an absence of benefit appears unlikely given the consistent preference for structured formats among clinicians reported in the oncology-focused literature [2, 11, 29]. Future studies with larger clinician samples will be needed to characterize oncologist preferences more precisely.

The present study deliberately addressed the complementary reader-centered question rather than serving as a formal accuracy benchmark. Nevertheless, previous benchmark results cannot establish the fidelity of the specific outputs evaluated here. Although each structured report was compared with its corresponding source report by both radiologists before survey administration, this author-led quality-control procedure did not use an external reference panel, blinded independent adjudicators, a prespecified error taxonomy, or quantitative accuracy endpoints.

Higher reader ratings for completeness and precision should therefore not be interpreted as evidence that the LLM conversion was free of hallucinations, omissions, unsupported additions, altered measurements, or laterality or site errors. Previous studies have demonstrated the feasibility of LLM-based conversion of radiology reports, but residual omissions, unsupported elements, confabulations, and clinically relevant misclassifications have also been reported, and performance varies across models, prompts, templates, languages, and clinical tasks [24, 30–34]. Accordingly, the present findings support the perceived value of radiologist-reviewed LLM-structured reports but do not establish the safety of autonomous report conversion.

The performance and reproducibility of LLM-based reporting applications depend on the model version, prompt, template, inference settings, language, and clinical task [34]. Although high fidelity has been reported in constrained radiology-report conversion tasks [24, 30–34], omissions, unsupported additions, confabulations, and clinically relevant misclassifications remain possible [31, 32].

Li and Chong described laterality as a potential failure mode in a multimodal radiology application [33]. Although that setting differs from the present text-only conversion task, it illustrates the need to include laterality and anatomic-site preservation in validation frameworks for LLM-assisted reporting. Li, Kim, and Yi further emphasized the influence of model versions and inference settings, the importance of transparent technical reporting, and the need for clinical validation [34].

The template versions reflected the staging and reporting standards applicable when the study was designed and initiated. Because the templates were finalized before participant recruitment and used unchanged throughout both survey phases, retrospectively replacing their criteria would no longer represent the reporting framework evaluated by the participants. Because only one synthetic report was evaluated for each cancer domain, the findings do not establish generalizability across all initial-staging, restaging, treatment-response, and surveillance scenarios. Inclusion of a prostate-cancer CT report assessed the presentation of information contained in that report and should not be interpreted as evidence that CT is sufficient for all prostate-cancer assessment tasks.

This study has limitations. First, the single-center convenience sample may reflect local reporting practices and limit generalizability to other institutions, languages, reader populations, and clinical workflows. Although recruitment was also advertised through professional networks outside the study institution, no external participant completed both survey phases. Independent multicenter studies with predefined site-level recruitment are needed to confirm the observed preferences. Second, because all participants evaluated free-text reports before structured reports, report format was confounded with the study period. The washout interval may have reduced direct recall, but cannot exclude familiarity, learning, expectancy, or other temporal effects. The findings should therefore be interpreted as within-participant preferences observed under a fixed implementation sequence rather than as an order-independent causal effect of report format. Future confirmatory studies should use randomized, counterbalanced sequence allocation. Third, participants could not be blinded to the visibly different report formats. Neutral framing of the participant information, concealment of the planned comparison and LLM origin during Phase 1, and separate presentation of the formats were intended to reduce expectancy and immediate comparison, but novelty and demand effects cannot be excluded. The study did not directly assess the influence of format awareness on participant ratings. The absence of a significant difference in readability and the concentration of improvements in organization-dependent items is not consistent with a completely indiscriminate increase in Phase 2 ratings. However, this response pattern cannot exclude expectancy or order effects. Fourth, the reports were presented in a fixed order. Because only one report represented each cancer domain, case-specific, domain-specific, and position effects cannot be separated fully, and domain-stratified findings should be regarded as exploratory. Although participants were instructed to evaluate the reports independently, independent completion of the unsupervised online questionnaires could not be technically verified. Fifth, the sample size of 66 paired participants limited the precision of subgroup analyses. The participating radiologists were relatively early in their careers, and the oncologist subgroup was small. The preferences of senior radiologists and highly subspecialized oncologists may therefore be underrepresented. Finally, the study assessed subjective reader perceptions rather than formal source-to-output fidelity or objective clinical outcomes. The structured reports underwent author-led quality control before survey administration, but no external reference panel, prespecified error taxonomy, or quantitative accuracy endpoints were used. Higher ratings for perceived completeness or precision should therefore not be interpreted as evidence that the conversion was free of hallucinations, omissions, unsupported additions, altered measurements, or laterality or site errors. Future studies should combine systematic content-concordance testing with randomized multicenter evaluation of reading time, clinical decision-making, and patient-relevant outcomes.

In conclusion, LLM-structured oncologic CT reports received higher ratings than conventional free-text reports across most evaluated dimensions, particularly for information findability, recommendation clarity, and clinical utility. The findings provide reader-centered evidence concerning quality-controlled LLM-structured reports but do not establish conversion accuracy or the safety of autonomous use. Formal source-to-output validation and prospectively planned multicenter assessment remain necessary before clinical implementation.

## Electronic supplementary material

Below is the link to the electronic supplementary material.

Supplementary Material 1

## Data Availability

Data generated in this study are available from the corresponding author upon request.
