# Supplementary material for: Multidisciplinary attitudes towards LLM-structured oncologic CT reports: a prospective fixed-sequence questionnaire study
Source: Cancer Imaging. 2026 Oct 8;26:140. doi: 10.1186/s40644-026-01137-2 (PMC13651054; doi:10.1186/s40644-026-01137-2)
Supplement: Supplementary file 1 [file 40644_2026_1137_MOESM1_ESM.docx]

**Supplemental Material**

**eTable 1.** Structured templates for each cancer domain in markdown format.

| **Cancer domain** | **Template** |
| --- | --- |
| Breast | ```markdown  Please extract the necessary information from the original text and fill in the relevant sections of this template. If specific information is not available, mark it as **N/A**.  ### 1. Clinical History  - Histology: [e.g., Invasive ductal carcinoma, NST, Grade 2 (Nottingham); Luminal B (ER+, PR+, HER2−, Ki-67 20%)]  - Diagnosis date: [MM/YYYY or "Not stated"]  - Tumor laterality: ☐ Right ☐ Left ☐ Bilateral ☐ Not stated  - Prior treatments: [e.g., surgery, chemotherapy (anastrozole), radiotherapy]  - Current / last treatment: [e.g., paclitaxel weekly, last given MM/YYYY]  - Time since last treatment: [e.g., "6 months", "Not stated"]  - Clinical question: ☐ Staging ☐ Restaging ☐ Therapy response ☐ Surveillance  - Comparison with prior imaging: [e.g., "Compared to CT from 10/2023", "No prior available"]  ### 2. Imaging Technique  - CT region: thorax, abdomen, and pelvis (± neck if clinically indicated)  - Acquisition: multislice contrast-enhanced CT  - Reconstructions: axial, coronal, sagittal MPR (± 3D)  - Contrast agents: [e.g., IV Imeron 400, oral contrast]  - Limitations: [e.g., "Streak artifact from port-a-cath", "None stated"]  ### 3. Primary Tumor (T)  > Note: CT has limited sensitivity for local T staging of breast cancer; MRI is preferred for local assessment.  - Tumor visible on CT: ☐ Yes ☐ No ☐ Not clearly visualized  - Tumor location: ☐ Right breast ☐ Left breast ☐ Not stated; quadrant: ☐ UOQ ☐ UIQ ☐ LOQ ☐ LIQ ☐ Central/subareolar  - Tumor size on CT (cm): [AP × ML × CC]  - Morphology: [e.g., irregular enhancing mass, satellite nodules]  - **T category by size (AJCC 8th):**  - ☐ T1 (≤ 20 mm) — pathologic subcategories if applicable: ☐ T1mi (≤ 1 mm) ☐ T1a (>1–5 mm) ☐ T1b (>5–10 mm) ☐ T1c (>10–20 mm)  - ☐ T2 (>20–50 mm)  - ☐ T3 (>50 mm, no T4 feature)  - ☐ T4 — see subcategories below  - **T4 subcategories (any one suffices):**  - ☐ T4a — chest wall involvement (ribs, intercostal or serratus anterior muscles; pectoralis alone does NOT qualify)  - ☐ T4b — skin edema (including peau d'orange), ulceration, or ipsilateral satellite skin nodules  - ☐ T4c — both T4a and T4b present  - ☐ T4d — inflammatory breast cancer (clinicopathologic diagnosis)  - Nipple involvement: ☐ Yes ☐ No  - Cutis involvement (non-T4b skin thickening): ☐ Yes ☐ No  - Satellite intramammary lesions (not skin): ☐ Present ☐ Absent  - Note: macroscopic dermal / skin satellite nodules → T4b (see above); microscopic dermal satellites alone do NOT qualify as T4b.  - MRI recommended for local staging: ☐ Yes ☐ No  - Free-text tumor comment: [Preserve nuances from source text]  ### 4. Regional Lymph Nodes (N)  > Regional stations: axillary (levels I–III), ipsilateral internal mammary, ipsilateral supraclavicular.  **Axillary nodes (levels I and II):**  - Suspicious: ☐ Yes ☐ No  - Largest short axis: [e.g., "11 mm"]  - Side / level: [e.g., "Right level I"]  - Movable vs fixed / matted: ☐ Movable ☐ Fixed or matted  - Change on follow-up: ☐ New ☐ Stable ☐ Increased ☐ Decreased ☐ N/A  **Level III (infraclavicular) nodes:**  - Suspicious: ☐ Yes ☐ No  - Details: [size, location]  **Internal mammary nodes:**  - Suspicious: ☐ Yes ☐ No  - Largest short axis: [cm or "Not specified"]  - Concurrent axillary involvement: ☐ Yes ☐ No  **Supraclavicular nodes (ipsilateral):**  - Suspicious: ☐ Yes ☐ No  - Details: [size, location]  **cN category (AJCC 8th, imaging / clinical):**  - ☐ cN0 — no regional LN metastases  - ☐ cN1 — movable ipsilateral level I–II axillary LN(s)  - ☐ cN2a — fixed or matted ipsilateral level I–II axillary LN(s)  - ☐ cN2b — ipsilateral internal mammary LN(s) WITHOUT axillary involvement  - ☐ cN3a — ipsilateral level III (infraclavicular) LN(s), with or without level I/II  - ☐ cN3b — ipsilateral internal mammary LN(s) WITH ipsilateral level I/II axillary involvement  - ☐ cN3c — ipsilateral supraclavicular LN(s)  - Free-text lymph node comment: [Optional]  ### 5. Distant Metastases (M)  > Note: Breast cancer uses only M0 / cM1 / pM1. There are no M1 subcategories.  - **M category:**  - ☐ M0 — no clinical or radiographic evidence of distant metastases  - ☐ cM1 — distant metastases by clinical or radiographic means  - ☐ pM1 — histologically proven distant metastases (>0.2 mm)  - **Sites of metastases (check all that apply):**  **Non-regional (distant) lymph nodes:**  - Suspicious: ☐ Yes ☐ No  - Location: [e.g., mediastinal, retroperitoneal, cervical non-supraclavicular]  - Largest short axis: [cm]  **Bone:**  - Osseous metastases: ☐ Yes ☐ No  - Location(s): ☐ Spine ☐ Pelvis ☐ Ribs ☐ Long bones ☐ Skull ☐ Other  - Lesion type: ☐ Lytic ☐ Sclerotic ☐ Mixed  - Risk of pathological fracture: ☐ Yes ☐ No  **Lung / pleura:**  - Suspicious lung lesions: ☐ Yes ☐ No  - Pattern: ☐ Single ☐ Multiple ☐ Disseminated  - Lobe(s): ☐ LUL ☐ LLL ☐ RUL ☐ RML ☐ RLL  - Largest lesion: [cm, location]  - Pleural effusion: ☐ None ☐ Present (☐ Benign ☐ Malignant)  - Change on follow-up: ☐ New ☐ Stable ☐ Increased ☐ Decreased  **Liver:**  - Suspicious lesions: ☐ Yes ☐ No  - Segment(s): [e.g., "Segment IVb"]  - Largest lesion: [cm]  - Change on follow-up: ☐ New ☐ Stable ☐ Increased ☐ Decreased  **Other sites:** [e.g., adrenal, peritoneum, ovary, CNS — recommend dedicated brain MRI if CNS disease suspected]  - Free-text metastasis comment: [Optional]  ### 6. Previous Imaging Comparison  - Prior study available: ☐ Yes (date: ________) ☐ No  - Summary of change per region:  \| Lesion type \| Change \|  \|--------------------\|--------------------------------------------------------\|  \| Primary tumor \| ☐ New ☐ Stable ☐ Increased ☐ Decreased ☐ Not described \|  \| Regional nodes \| ☐ New ☐ Stable ☐ Increased ☐ Decreased ☐ Not described \|  \| Lung metastases \| ☐ New ☐ Stable ☐ Increased ☐ Decreased ☐ Not described \|  \| Bone metastases \| ☐ New ☐ Stable ☐ Increased ☐ Decreased ☐ Not described \|  \| Liver lesions \| ☐ New ☐ Stable ☐ Increased ☐ Decreased ☐ Not described \|  ### 7. Additional Findings  - Relevant incidental findings: ☐ None ☐ Present (see below)  - ☐ Focal fatty liver  - ☐ Cholelithiasis  - ☐ Renal cysts  - ☐ Uterine myoma  - ☐ Other: ______________________  - Free-text comment: [If clinically relevant]  ### 8. TNM Summary  - **cT:** ☐ TX ☐ T1 (☐ T1mi ☐ T1a ☐ T1b ☐ T1c) ☐ T2 ☐ T3 ☐ T4a ☐ T4b ☐ T4c ☐ T4d  - **cN:** ☐ NX ☐ N0 ☐ N1 ☐ N2a ☐ N2b ☐ N3a ☐ N3b ☐ N3c  - **cM:** ☐ M0 ☐ cM1 ☐ pM1  ### 9. Conclusion  - ☐ No evidence of distant metastatic disease (M0)  - ☐ Metastatic disease identified  - Sites involved: [e.g., lungs, axillary lymph nodes, liver, bone]  - Comparison to prior: ☐ New ☐ Stable ☐ Increased ☐ Decreased  - ☐ Disease progression ☐ Stable disease ☐ Partial response ☐ Complete response  - Further imaging recommended: ☐ PET-CT ☐ Breast MRI ☐ Brain MRI ☐ Biopsy ☐ None  - **Summary:** [Brief overview]  ### 10. Internal Consistency Checklist  - ☐ All metastatic sites from text reflected in M section  - ☐ Impression matches TNM findings  - ☐ Progression / stability documented  - ☐ Prior comparison clearly stated  - ☐ Any ambiguous findings flagged for review  ``` |
| Colorectal | ```markdown  Please extract the necessary information from the original text and fill in the relevant sections of this template. If specific information is not available, mark it as **N/A**.  ### 1. Clinical History  - Histology: [e.g., moderately differentiated adenocarcinoma, G2]  - Diagnosis date: [MM/YYYY]  - Primary tumor location: ☐ Cecum ☐ Ascending colon ☐ Hepatic flexure ☐ Transverse colon ☐ Splenic flexure ☐ Descending colon ☐ Sigmoid colon ☐ Rectosigmoid ☐ Rectum ☐ Not stated  - Prior treatments: [e.g., hemicolectomy MM/YYYY, FOLFOX chemotherapy]  - Current / last treatment: [e.g., FOLFIRI + bevacizumab, last MM/YYYY]  - Time since last treatment: [duration]  - Clinical question: ☐ Staging ☐ Restaging ☐ Therapy response ☐ Surveillance  - Comparison with prior imaging: ☐ Yes (date: __________) ☐ No  - Disease status at last imaging: ☐ Stable ☐ Progressive ☐ Partial response ☐ Complete response ☐ Not stated  ### 2. Imaging Technique  - CT region: thorax, abdomen, and pelvis  - Acquisition: multislice contrast-enhanced CT  - Reconstructions: axial, coronal, sagittal MPR  - Contrast agents: [e.g., IV Imeron 400; oral and/or rectal contrast]  - Limitations: [e.g., "Beam-hardening artifact from hip prostheses", "None stated"]  ### 3. Primary Tumor (T)  > Note: CT has limited sensitivity for local T staging, especially T1 vs T2. Rectal MRI is preferred for rectal cancer local staging.  - Tumor visible on CT: ☐ Yes ☐ No ☐ Not clearly visualized  - Tumor location: _________________  - Tumor size (cm): _________________  - Morphology: ☐ Circumscribed ☐ Irregular ☐ Infiltrative ☐ Napkin-ring / annular ☐ Other: _________  - **T category (AJCC 8th):**  - ☐ T1 — invades submucosa (rarely assessable on CT)  - ☐ T2 — invades muscularis propria (limited CT assessability)  - ☐ T3 — extramural invasion into pericolorectal tissues  - ☐ T4a — penetrates visceral peritoneum  - ☐ T4b — invades or is adherent to adjacent organs / structures  - Associated findings: ☐ Bowel obstruction ☐ Perforation ☐ Fistula ☐ Abscess ☐ None  - MRI recommended (rectal cancer local staging): ☐ Yes ☐ No ☐ N/A  - Free-text tumor comment: _________________  ### 4. Regional Lymph Nodes (N)  > Regional LNs: pericolic, perirectal / mesorectal, and nodes along the named vascular pedicles (ileocolic, right/middle/left colic, inferior mesenteric artery/vein, sigmoidal, superior rectal).  - Suspicious lymphadenopathy: ☐ Yes ☐ No  - Location: ☐ Pericolic ☐ Perirectal / mesorectal ☐ Along named vascular pedicle (specify: ________) ☐ Other: _________________  - Number of suspicious nodes on imaging: _________________  - Largest short axis: _________ cm  - Tumor deposits (discrete nodules without residual nodal tissue): ☐ Yes ☐ No  - Change on follow-up: ☐ New ☐ Stable ☐ Increased ☐ Decreased  - **N category (AJCC 8th, imaging estimate):**  - ☐ N0 — no regional LN metastases  - ☐ N1a — 1 positive regional LN  - ☐ N1b — 2–3 positive regional LNs  - ☐ N1c — tumor deposit(s) in subserosa / mesentery / pericolorectal tissue without positive regional LNs  - ☐ N2a — 4–6 positive regional LNs  - ☐ N2b — ≥7 positive regional LNs  - Free-text lymph node comment: _________________  ### 5. Distant Metastases (M)  - Number of organs / sites involved: ☐ 0 ☐ 1 ☐ ≥ 2  - Peritoneal involvement: ☐ Yes ☐ No  - **M category (AJCC 8th):**  - ☐ M0 — no distant metastases  - ☐ M1a — metastasis to one organ or site, without peritoneal involvement  - ☐ M1b — metastases to two or more organs or sites, without peritoneal involvement  - ☐ M1c — peritoneal metastasis, with or without involvement of other organs or sites  - **Sites of metastases (check all that apply):**  ☐ Liver  - Number of lesions: ☐ Single ☐ Multiple  - Segment(s): _________  - Largest lesion: _________ cm  - Change on follow-up: ☐ New ☐ Stable ☐ Increased ☐ Decreased  ☐ Lung  - Pattern: ☐ Single ☐ Multiple  - Lobe(s): ☐ LUL ☐ LLL ☐ RUL ☐ RML ☐ RLL  - Largest lesion: _________ cm  - Change on follow-up: ☐ New ☐ Stable ☐ Increased ☐ Decreased  ☐ Non-regional lymph nodes (e.g., retroperitoneal, mediastinal, cervical)  - Location: _________  - Largest short axis: _________ cm  ☐ Bone  - Location(s): ☐ Spine ☐ Pelvis ☐ Ribs ☐ Long bones ☐ Other: _________  - Lesion type: ☐ Sclerotic ☐ Lytic ☐ Mixed  - Risk of pathological fracture: ☐ Yes ☐ No  ☐ Peritoneum  - Distribution: ☐ Localized ☐ Diffuse  - Ascites: ☐ Yes ☐ No  ☐ Ovary / other: _________  - Free-text metastasis comment: _________________  ### 6. Previous Imaging Comparison  - Prior study available: ☐ Yes (date: __________) ☐ No  - Summary of change per region:  \| Lesion type \| Change \|  \|---------------------\|--------------------------------------------------------\|  \| Primary tumor \| ☐ New ☐ Stable ☐ Increased ☐ Decreased ☐ Not described \|  \| Regional lymph nodes\| ☐ New ☐ Stable ☐ Increased ☐ Decreased ☐ Not described \|  \| Liver metastases \| ☐ New ☐ Stable ☐ Increased ☐ Decreased ☐ Not described \|  \| Lung metastases \| ☐ New ☐ Stable ☐ Increased ☐ Decreased ☐ Not described \|  \| Peritoneum \| ☐ New ☐ Stable ☐ Increased ☐ Decreased ☐ Not described \|  ### 7. Additional Findings  - Relevant incidental findings: ☐ Present ☐ Absent  - Description: _________________  ### 8. TNM Summary  - **cT:** ☐ TX ☐ T1 ☐ T2 ☐ T3 ☐ T4a ☐ T4b  - **cN:** ☐ NX ☐ N0 ☐ N1a ☐ N1b ☐ N1c ☐ N2a ☐ N2b  - **cM:** ☐ M0 ☐ M1a ☐ M1b ☐ M1c  ### 9. Conclusion  - ☐ No evidence of distant metastatic disease (M0)  - ☐ Metastatic disease identified  - Sites involved: _________________  - Comparison to prior: ☐ New ☐ Stable ☐ Increased ☐ Decreased  - ☐ Disease progression ☐ Stable disease ☐ Partial response ☐ Complete response  - Further imaging recommended: ☐ Rectal MRI ☐ PET-CT ☐ None  - **Summary:** [Brief overview]  ### 10. Internal Consistency Checklist  - ☐ All metastatic sites from text reflected in M section  - ☐ Impression matches TNM findings  - ☐ Progression / stability documented  - ☐ Prior comparison clearly stated  - ☐ Any ambiguous findings flagged for review  ``` |
| Hepatocellular carcinoma | ```markdown  Please extract the necessary information from the original text and fill in the relevant sections of this template. If specific information is not available, mark it as **N/A**.  ### 1. Clinical History  - Diagnosis: [e.g., hepatocellular carcinoma, status post TACE MM/YYYY]  - Underlying liver disease: [e.g., cirrhosis Child-Pugh A, chronic hepatitis C, NASH]  - Prior treatments: [e.g., TACE, TIPS, ablation, resection, atezolizumab/bevacizumab]  - Current / last treatment: [e.g., TACE MM/YYYY]  - Time since last treatment: [duration]  - Clinical question: ☐ Staging ☐ Restaging ☐ Therapy response ☐ Surveillance  - Comparison with prior imaging: [date or "Not available"]  ### 2. Imaging Technique  - CT region: thorax, abdomen, and pelvis (multiphasic liver CT: unenhanced, late arterial, portal venous, ± delayed)  - Acquisition: multislice contrast-enhanced CT with multiphase liver protocol  - Reconstructions: axial, coronal, sagittal MPR (± 3D)  - Contrast agents: [e.g., IV Imeron 400, oral contrast]  - Limitations: [e.g., "Respiratory artifacts", "None stated"]  ### 3. Primary Tumor (T)  - Hepatic tumor visible on CT: ☐ Yes ☐ No  - Number of lesions: ☐ Single ☐ Multiple  - Maximum tumor dimension (largest lesion): _________ cm  - Imaging-evident vascular invasion: ☐ None ☐ Segmental / sectoral branch (peripheral) ☐ Major branch of the portal vein (right, left, or main) ☐ Major branch of the hepatic vein (right, middle, or left, or IVC at its confluence) ☐ IVC / right atrium  - Change from prior study (date: __________): ☐ New ☐ Stable ☐ Increased ☐ Decreased  - **T category (AJCC 8th):**  - ☐ T1a — solitary tumor ≤ 2 cm (with OR without vascular invasion)  - ☐ T1b — solitary tumor > 2 cm WITHOUT vascular invasion  - ☐ T2 — solitary tumor > 2 cm WITH vascular invasion, OR multiple tumors none > 5 cm  - ☐ T3 — multiple tumors, at least one > 5 cm  - ☐ T4 — tumor(s) of any size involving a major branch of the portal or hepatic vein, OR direct invasion of adjacent organs other than the gallbladder, OR perforation of visceral peritoneum  - Free-text tumor comment: [Narrative summary of complex local invasion if needed]  ### 4. LI-RADS v2018 Categorization (CT/MRI)  For each observation:  - Observation #: [1 / 2 / 3 / 4 / 5 …]  - Location: [segment I / II / III / IVa / IVb / V / VI / VII / VIII]  - Size: [ ] × [ ] mm (image [ ], series [ ])  - Major features: ☐ Arterial phase hyperenhancement (APHE) ☐ Non-peripheral "washout" ☐ Enhancing capsule ☐ Threshold growth  - Ancillary features favoring malignancy / HCC: [list]  - Tumor in vein (TIV): ☐ No ☐ Yes (describe extent and location)  - **LI-RADS category:** ☐ LR-NC ☐ LR-1 ☐ LR-2 ☐ LR-3 ☐ LR-4 ☐ LR-5 ☐ LR-TIV ☐ LR-M ☐ LR-TR (treatment response: viable / equivocal / nonviable)  ### 5. Regional Lymph Nodes (N)  > Regional LNs for HCC: hilar (hepatoduodenal ligament), periportal, hepatic artery, inferior phrenic, and caval nodes.  - Lymph node involvement: ☐ No suspicious lymphadenopathy (N0) ☐ Suspicious lymphadenopathy (N1)  - Largest node size: _________ mm short axis; location: _________  - Change from prior (date: __________): ☐ New ☐ Stable ☐ Increased ☐ Decreased  - **N category:** ☐ NX ☐ N0 ☐ N1  - Free-text LN comment: [e.g., "Multiple necrotic lymph nodes causing vascular compression"]  ### 6. Distant Metastases (M)  > Note: HCC uses only M0 / M1. There are no M1 subcategories.  - **M category:** ☐ M0 ☐ M1  - **Sites of metastases (check all that apply):**  ☐ Non-regional lymph nodes  - Location: _________  - Largest short axis: _________ mm  ☐ Lungs  - Pattern: ☐ Single ☐ Multiple  - Lobe(s): _________  - Largest lesion: _________ cm  - Change from prior: ☐ New ☐ Stable ☐ Increased ☐ Decreased  ☐ Bone  - Location(s): ☐ Spine ☐ Pelvis ☐ Ribs ☐ Long bones ☐ Other: _________  - Lesion type: ☐ Lytic ☐ Sclerotic ☐ Mixed  - Risk of pathological fracture: ☐ Yes ☐ No  ☐ Adrenal glands  - Side: ☐ Left (size: _________ cm) ☐ Right (size: _________ cm) ☐ Bilateral  - Change from prior: ☐ New ☐ Stable ☐ Increased ☐ Decreased  ☐ Peritoneum / other sites: _________  - Free-text metastasis comment: [Optional]  ### 7. Previous Imaging Comparison  - Prior study available: ☐ Yes (date: __________) ☐ No  - Summary of change per region:  \| Lesion type \| Change \|  \|--------------------------\|--------------------------------------------------------\|  \| Primary hepatic tumor(s) \| ☐ New ☐ Stable ☐ Increased ☐ Decreased ☐ Not described \|  \| Regional lymph nodes \| ☐ New ☐ Stable ☐ Increased ☐ Decreased ☐ Not described \|  \| Vascular invasion \| ☐ New ☐ Stable ☐ Increased ☐ Decreased ☐ Not described \|  \| Distant metastases \| ☐ New ☐ Stable ☐ Increased ☐ Decreased ☐ Not described \|  ### 8. Additional Findings  - Relevant incidental findings (non-oncologic): _________  - Portal hypertension features: ☐ Splenomegaly ☐ Varices ☐ Ascites ☐ Recanalized paraumbilical vein ☐ None  - Biliary dilatation: ☐ Yes ☐ No  ### 9. TNM Summary and Conclusion  - **cT:** ☐ TX ☐ T1a ☐ T1b ☐ T2 ☐ T3 ☐ T4  - **cN:** ☐ NX ☐ N0 ☐ N1  - **cM:** ☐ M0 ☐ M1  - LI-RADS assessment summary:  - Observation 1: [LR-category]  - Observation 2: [LR-category]  - Observation 3: [LR-category]  - Impression:  - ☐ No evidence of distant metastatic disease (M0)  - ☐ Metastatic disease identified at: _________  - ☐ Disease progression ☐ Stable disease ☐ Partial response ☐ Complete response  - **Summary:** [Brief overview]  ### 10. Internal Consistency Checklist  - ☐ All observations receive LI-RADS categorization  - ☐ Vascular invasion status consistent between Section 3 and LI-RADS TIV  - ☐ Impression matches TNM findings  - ☐ Progression / stability documented  - ☐ Prior comparison clearly stated  ``` |
| Prostate | ```markdown  Please extract the necessary information from the original text and fill in the relevant sections of this template. If specific information is not available, mark it as **N/A**.  ### 1. Clinical History  - Diagnosis: [e.g., prostate adenocarcinoma, ISUP Grade Group 4, diagnosed MM/YYYY]  - PSA at diagnosis: _________ ng/mL; most recent PSA: _________ ng/mL (date: __________)  - Presentation: [e.g., progressive pain, PSA rise, incidental]  - Last staging: [duration since last staging]  - Clinical question: ☐ Staging ☐ Restaging ☐ Therapy response ☐ Surveillance  - Comparison with prior study: [date]  ### 2. Imaging Technique  > Note: CT has low sensitivity for local T staging. Multiparametric prostate MRI is preferred for local staging; PSMA PET-CT is preferred for occult nodal / distant metastases.  - CT region: thorax, abdomen, and pelvis  - Acquisition: multislice contrast-enhanced CT  - Reconstructions: axial, coronal, sagittal MPR (± 3D)  - Contrast agents: [e.g., IV Imeron 400, oral contrast]  - Limitations: [e.g., "Beam-hardening artifact from bilateral hip prostheses"]  ### 3. Primary Tumor (T)  - Prostate appearance on CT: ☐ Normal ☐ Enlarged ☐ Heterogeneous enhancement ☐ Not assessable  - Extraprostatic extension: ☐ Absent ☐ Present (T3a)  - Seminal vesicle invasion: ☐ Absent ☐ Present (T3b)  - Invasion of adjacent structures: ☐ None ☐ External sphincter ☐ Rectum ☐ Bladder ☐ Levator muscles ☐ Pelvic wall (any → T4)  - **cT category (AJCC 8th):**  - ☐ cTX — not assessable  - ☐ cT1 — clinically inapparent, detected by TURP or needle biopsy (not CT-assessable)  - ☐ cT2a — tumor involves ≤ one half of one side of the prostate  - ☐ cT2b — tumor involves > one half of one side, but not both sides  - ☐ cT2c — tumor involves both sides  - ☐ cT3a — extraprostatic extension (unilateral or bilateral) or microscopic bladder-neck invasion  - ☐ cT3b — tumor invades the seminal vesicle(s)  - ☐ cT4 — tumor fixed or invading external sphincter, rectum, bladder, levator muscles, or pelvic wall  - Free-text tumor comment: [Narrative summary if needed]  ### 4. Regional Lymph Nodes (N)  > Regional LNs (AJCC 8th): pelvic nodes below the common iliac artery bifurcation — periprostatic, obturator, internal iliac (hypogastric), external iliac, and sacral (presacral, lateral sacral, promontory).  - Suspicious lymphadenopathy: ☐ No (N0) ☐ Yes (N1)  - Location(s): ☐ Obturator ☐ External iliac ☐ Internal iliac (hypogastric) ☐ Sacral / presacral ☐ Periprostatic ☐ Other: ________  - Number: ☐ Single ☐ Multiple  - Largest short axis: _________ cm, location: _________  - **N category:** ☐ NX ☐ N0 ☐ N1  - Change from prior: ☐ New ☐ Stable ☐ Increased ☐ Decreased  ### 5. Distant Metastases (M)  - **M category (AJCC 8th):**  - ☐ M0 — no distant metastases  - ☐ M1a — non-regional lymph node(s) (above the common iliac artery bifurcation)  - ☐ M1b — bone metastasis/es  - ☐ M1c — other site(s), with or without bone disease  **Non-regional lymph nodes (M1a):**  - Suspicious: ☐ Yes ☐ No  - Location: ☐ Common iliac ☐ Retroperitoneal ☐ Mediastinal ☐ Supraclavicular ☐ Other: _________  - Largest short axis: _________ cm  **Bone (M1b):**  - Osseous metastases: ☐ Yes ☐ No  - Location(s): ☐ Spine ☐ Pelvis ☐ Ribs ☐ Long bones ☐ Skull ☐ Other: _________  - Lesion type: ☐ Sclerotic / osteoblastic ☐ Lytic ☐ Mixed  - Risk of pathological fracture: ☐ Yes ☐ No  **Visceral / other sites (M1c):**  - Lungs: ☐ Single ☐ Multiple; largest _________ cm, lobe: _________  - Liver: ☐ Single ☐ Multiple; largest _________ cm, segment: _________  - Other: _________  - Free-text metastasis comment: [Optional]  ### 6. Previous Imaging Comparison  - Prior study available: ☐ Yes (date: __________) ☐ No  - Summary of change per region:  \| Lesion type \| Change \|  \|---------------------------\|--------------------------------------------------------\|  \| Primary tumor / prostate \| ☐ New ☐ Stable ☐ Increased ☐ Decreased ☐ Not described \|  \| Regional lymph nodes \| ☐ New ☐ Stable ☐ Increased ☐ Decreased ☐ Not described \|  \| Non-regional lymph nodes \| ☐ New ☐ Stable ☐ Increased ☐ Decreased ☐ Not described \|  \| Bone metastases \| ☐ New ☐ Stable ☐ Increased ☐ Decreased ☐ Not described \|  \| Visceral metastases \| ☐ New ☐ Stable ☐ Increased ☐ Decreased ☐ Not described \|  ### 7. Additional Findings  - Relevant incidental findings: _________  ### 8. TNM Summary  - **cT:** ☐ TX ☐ T1 ☐ T2a ☐ T2b ☐ T2c ☐ T3a ☐ T3b ☐ T4  - **cN:** ☐ NX ☐ N0 ☐ N1  - **cM:** ☐ M0 ☐ M1a ☐ M1b ☐ M1c  ### 9. Conclusion  - ☐ No evidence of distant metastatic disease (M0)  - ☐ Metastatic disease identified  - Sites involved: [e.g., pelvic lymph nodes, bones, visceral organs]  - Comparison to prior: ☐ New ☐ Stable ☐ Increased ☐ Decreased  - Further imaging recommended: ☐ Multiparametric prostate MRI ☐ PSMA PET-CT ☐ Bone scan ☐ None  - **Summary:** [Brief narrative]  ### 10. Internal Consistency Checklist  - ☐ All metastatic sites from text reflected in M section  - ☐ Regional vs non-regional LN distinction observed (iliac bifurcation as boundary)  - ☐ Impression matches TNM findings  - ☐ Progression / stability documented  - ☐ Prior comparison clearly stated  ``` |
| Lung | ```markdown  Please extract the necessary information from the original text and fill in the relevant sections of this template. If specific information is not available, mark it as **N/A**.  ### 1. Clinical History  - Diagnosis: [e.g., non-small cell lung cancer (adenocarcinoma), diagnosed MM/YYYY; or "small cell lung cancer" — note that SCLC uses the same TNM system since AJCC 8th]  - Molecular / biomarker status: [e.g., EGFR, ALK, ROS1, PD-L1 %]  - Prior treatments: [e.g., chemotherapy (cisplatin + pemetrexed), radiotherapy, immunotherapy]  - Current / last treatment: [e.g., atezolizumab, last given MM/YYYY]  - Time since last treatment: [duration]  - Clinical question: ☐ Staging ☐ Restaging ☐ Therapy response ☐ Surveillance  - Comparison with prior study: [date]  ### 2. Imaging Technique  - CT region: thorax, abdomen, and pelvis (± neck if clinically indicated for supraclavicular assessment)  - Acquisition: multislice contrast-enhanced CT  - Reconstructions: axial, coronal, sagittal MPR (± 3D); lung and soft-tissue windows  - Contrast agents: [e.g., IV Imeron 400, oral contrast]  - Limitations: [e.g., "Motion artifact affecting lung apices"]  ### 3. Primary Tumor (T)  - Tumor visible on CT: ☐ Yes ☐ No  - Side: ☐ Right lung ☐ Left lung  - Lobe: ☐ RUL ☐ RML ☐ RLL ☐ LUL ☐ Lingula ☐ LLL  - Maximum tumor dimension (solid component, lung window, largest axis): _________ cm  - Change from prior (date: __________): ☐ New ☐ Stable ☐ Increased ☐ Decreased  - **T category by size (AJCC 8th; use when no other T-upstaging feature):**  - ☐ T1a — ≤ 1 cm  - ☐ T1b — > 1–2 cm  - ☐ T1c — > 2–3 cm  - ☐ T2a — > 3–4 cm  - ☐ T2b — > 4–5 cm  - ☐ T3 — > 5–7 cm  - ☐ T4 — > 7 cm  - **Feature-based upstaging (upgrades regardless of size):**  - ☐ T2 feature: involves main bronchus (regardless of distance from carina, but NOT carina itself), OR invades visceral pleura, OR atelectasis / obstructive pneumonitis extending to hilum or the entire lung  - ☐ T3 feature: invades parietal pleura (PL3), chest wall (including superior sulcus / Pancoast tumor), phrenic nerve, or parietal pericardium; OR separate tumor nodule in the SAME lobe  - ☐ T4 feature: invades diaphragm, mediastinum, heart, great vessels, trachea, carina, recurrent laryngeal nerve, esophagus, or vertebral body; OR separate tumor nodule in a DIFFERENT ipsilateral lobe  - Free-text tumor comment: [Narrative summary of complex local invasion]  ### 4. Regional Lymph Nodes (N)  > All intrathoracic nodes (hilar, mediastinal, subcarinal) AND scalene / supraclavicular nodes are regional for lung cancer.  - ☐ N0 — no regional LN metastasis  - ☐ N1 — ipsilateral peribronchial, hilar, or intrapulmonary (stations 10–14)  - ☐ N2 — ipsilateral mediastinal and/or subcarinal (stations 2–9 ipsilateral, 7)  - ☐ N3 — contralateral mediastinal or hilar, OR ipsilateral / contralateral scalene or supraclavicular (stations 1 and any contralateral 2–14)  - Largest node short axis: _________ mm; station(s): _________  - Change from prior (date: __________): ☐ New ☐ Stable ☐ Increased ☐ Decreased  - Free-text LN comment: [e.g., "Multiple necrotic mediastinal nodes with vascular compression"]  ### 5. Distant Metastases (M)  > Important: mediastinal, hilar, scalene, and supraclavicular nodes are REGIONAL (N category), NOT distant metastases. Record them in Section 4.  **Intrathoracic metastases (M1a):**  - ☐ None  - ☐ Contralateral lung nodule(s): ☐ Single ☐ Multiple; lobe(s): _________; largest _________ cm  - ☐ Pleural metastasis: ☐ Nodules ☐ Malignant pleural effusion  - ☐ Pericardial metastasis: ☐ Nodules ☐ Malignant pericardial effusion  - Change from prior: ☐ New ☐ Stable ☐ Increased ☐ Decreased  **Extrathoracic metastases:**  - ☐ None  - ☐ Single extrathoracic metastasis in a single organ (M1b):  - Site: ☐ Non-regional LN (e.g., cervical non-supraclavicular, axillary, abdominal, retroperitoneal) ☐ Bone ☐ Liver ☐ Adrenal ☐ Brain ☐ Other: _________  - Location / largest lesion: _________  - ☐ Multiple extrathoracic metastases (M1c): single or multiple organ systems  - Sites involved (check all that apply):  - ☐ Non-regional LN: _________  - ☐ Bone: location _________; type ☐ Sclerotic ☐ Lytic ☐ Mixed; fracture risk ☐ Yes ☐ No  - ☐ Liver: _________  - ☐ Adrenal: ☐ Left (size: _________ cm) ☐ Right (size: _________ cm) ☐ Bilateral  - ☐ Brain (dedicated brain MRI / CT recommended if not yet performed)  - ☐ Other: _________  - Change from prior: ☐ New ☐ Stable ☐ Increased ☐ Decreased  - Free-text metastasis comment: [Optional]  ### 6. Previous Imaging Comparison  - Prior study available: ☐ Yes (date: __________) ☐ No  - Summary of change per region:  \| Lesion type \| Change \|  \|---------------------------\|--------------------------------------------------------\|  \| Primary tumor \| ☐ New ☐ Stable ☐ Increased ☐ Decreased ☐ Not described \|  \| Regional lymph nodes \| ☐ New ☐ Stable ☐ Increased ☐ Decreased ☐ Not described \|  \| Intrathoracic metastases \| ☐ New ☐ Stable ☐ Increased ☐ Decreased ☐ Not described \|  \| Extrathoracic metastases \| ☐ New ☐ Stable ☐ Increased ☐ Decreased ☐ Not described \|  ### 7. Additional Findings  - Incidental findings (non-oncologic): [e.g., emphysema, coronary calcifications, degenerative spine disease]  ### 8. TNM Summary  - **cT:** ☐ TX ☐ T1a ☐ T1b ☐ T1c ☐ T2a ☐ T2b ☐ T3 ☐ T4  - **cN:** ☐ NX ☐ N0 ☐ N1 ☐ N2 ☐ N3  - **cM:** ☐ M0 ☐ M1a ☐ M1b ☐ M1c  ### 9. Conclusion  - ☐ No evidence of distant metastatic disease (M0)  - ☐ Metastatic disease identified  - Sites involved: _________  - Comparison to prior: ☐ New ☐ Stable ☐ Increased ☐ Decreased  - ☐ Disease progression ☐ Stable disease ☐ Partial response ☐ Complete response  - Further imaging recommended: ☐ Dedicated brain MRI / CT ☐ PET-CT ☐ Pleural biopsy ☐ None  - **Summary:** [Brief overview]  ### 10. Internal Consistency Checklist  - ☐ All metastatic sites from text reflected in M section  - ☐ Mediastinal / hilar / scalene / supraclavicular nodes correctly placed under N (not M)  - ☐ Contralateral lung nodules and pleural / pericardial disease classified as M1a (not N)  - ☐ Single vs multiple extrathoracic metastases distinction (M1b vs M1c) applied  - ☐ Impression matches TNM findings  ``` |

**eTable 2.** Characteristics of participants who did not complete phase 2.

| **Characteristic** | **Dropouts (n=4)** |
| --- | --- |
| Gender, no. (%) |  |
| Female | 2 (50.0) |
| Male | 2 (50.0) |
| Age, mean (SD), y | 26.3 (1.0) |
| Experience, mean (SD), y | 2.0 (2.0) |
| Expertise group, no. (%) |  |
| Radiologists | 0 (0.0) |
| Technologists | 1 (25.0) |
| Oncologists | 2 (50.0) |
| Students | 1 (25.0) |

**Note:** Abbreviation: SD, standard deviation.

**Figure S1.** Gantt diagrams illustrating item responses for the readability and comprehension domain for radiologists (n=22): A) readability (Q1), B) clarity (Q2), C) findability (Q3).

**Figure S2.** Gantt diagrams illustrating item responses for the readability and comprehension domain for radiology technologists (n=12): A) readability (Q1), B) clarity (Q2), C) findability (Q3).

**Figure S3.** Gantt diagrams illustrating item responses for the readability and comprehension domain for oncologists (n=8): A) readability (Q1), B) clarity (Q2), C) findability (Q3).

**Figure S4.** Gantt diagrams illustrating item responses for the readability and comprehension domain for medical students (n=24): A) readability (Q1), B) clarity (Q2), C) findability (Q3).

**Figure S5.** Gantt diagrams illustrating item responses for the oncologic information quality domain for radiologists (n=22): A) oncologic clarity (Q4), B) oncologic structure/organization (Q5), C) oncologic comprehensibility (Q6), D) oncologic completeness (Q7), E) oncologic precision (Q8).

**Figure S6.** Gantt diagrams illustrating item responses for the oncologic information quality domain for radiology technologists (n=12): A) oncologic clarity (Q4), B) oncologic structure/organization (Q5), C) oncologic comprehensibility (Q6), D) oncologic completeness (Q7), E) oncologic precision (Q8).

**Figure S7.** Gantt diagrams illustrating item responses for the oncologic information quality domain for oncologists (n=8): A) oncologic clarity (Q4), B) oncologic structure/organization (Q5), C) oncologic comprehensibility (Q6), D) oncologic completeness (Q7), E) oncologic precision (Q8).

**Figure S8.** Gantt diagrams illustrating item responses for the oncologic information quality domain for medical students (n=24): A) oncologic clarity (Q4), B) oncologic structure/organization (Q5), C) oncologic comprehensibility (Q6), D) oncologic completeness (Q7), E) oncologic precision (Q8).

**Figure S9.** Gantt diagrams illustrating item responses for the clinical utility domain for radiologists (n=22): A) clinical utility (Q9), B) clinical decision support (Q10), C) recommendation clarity (Q11), D) patient communication suitability (Q12).

**Figure S10.** Gantt diagrams illustrating item responses for the clinical utility domain for radiology technologists (n=12): A) clinical utility (Q9), B) clinical decision support (Q10), C) recommendation clarity (Q11), D) patient communication suitability (Q12).

**Figure S11.** Gantt diagrams illustrating item responses for the clinical utility domain for oncologists (n=8): A) clinical utility (Q9), B) clinical decision support (Q10), C) recommendation clarity (Q11), D) patient communication suitability (Q12).

**Figure S12.** Gantt diagrams illustrating item responses for the clinical utility domain for medical students (n=24): A) clinical utility (Q9), B) clinical decision support (Q10), C) recommendation clarity (Q11), D) patient communication suitability (Q12).
